# Multi-Functional Development and Utilization of Rapeseed: Comprehensive Analysis of the Nutritional Value of Rapeseed Sprouts

**DOI:** 10.3390/foods11060778

**Published:** 2022-03-08

**Authors:** Zelin Xiao, Yuying Pan, Chao Wang, Xiongcai Li, Yiqing Lu, Ze Tian, Lieqiong Kuang, Xinfa Wang, Xiaoling Dun, Hanzhong Wang

**Affiliations:** 1 Laboratory of Biology and Genetic Improvement of Oil Crops, Oil Crops Research Institute of the Chinese Academy of Agricultural Sciences/Key, Ministry of Agriculture and Rural Affairs, Wuhan 430062, China; xzl20202022@163.com (Z.X.); pyy960324@163.com (Y.P.); wangchao05360536@163.com (C.W.); 12016149@zju.edu.cn (Y.L.); tianze0825@163.com (Z.T.); kuanglieqiong@163.com (L.K.); wangxinfa@caas.cn (X.W.); wanghz@oilcrops.cn (H.W.); 2 Hubei Hongshan Laboratory, Wuhan 430070, China; 3 Xiangyang Agricultural Technology Extension Center, Xiangyang 441021, China; xfnjtgzx@163.com

**Keywords:** rapeseed, sprout, vitamin E, glucosinolates, selenium

## Abstract

Rapeseed is the third largest oil crop in the world and the largest oil crop in China. The multi-functional development and utilization of rapeseed is an effective measure for the high-quality development of rapeseed industry in China. In this study, several basic nutrients of eight rapeseed sprouts and five bean sprouts (3–5 varieties each) were determined, including sugar, crude protein, crude fiber, vitamin E, minerals, fatty acids, amino acids, and glucosinolates. Data analysis revealed that compared with bean sprouts, rapeseed sprouts were nutritionally balanced and were richer in active nutrients such as glucose, magnesium, selenium, vitamin E, and glucosinolate. Moreover, rapeseed sprouts exhibited reasonable amino acid composition and abundant unsaturated fatty acids (accounting for 90.32% of the total fatty acids). All these results indicated the potential of rapeseed sprout as a functional vegetable. Subsequently, three dominant nutrients including vitamin E, glucosinolate, and selenium were investigated in seeds and sprouts of 44 *B. napus L.* varieties. The results showed that germination raised the ratio of α-tocopherol/γ-tocopherol from 0.53 in seeds to 9.65 in sprouts, greatly increasing the content of α-tocopherol with the strongest antioxidant activity among the eight isomers of vitamin E. Furthermore, germination promoted the conversion and accumulation of glucosinolate components, especially, glucoraphanin with strong anti-cancer activity with its proportion increased from 1.06% in seeds to 1.62% in sprouts. In addition, the contents of selenium, vitamin E, and glucosinolate in rapeseed sprouts were highly correlated with those in seeds. Furthermore, these three dominant nutrients varied greatly within *B. napus* varieties, indicating the great potential of rapeseed sprouts to be further bio-enhanced. Our findings provide reference for the multi-purpose development and utilization of rapeseed, lay a theoretical foundation for the development of rapeseed sprout into a functional vegetable, and provide a novel breeding direction.

## 1. Introduction

Rapeseed is not only the world’s third largest oil crop, but also the most important oil crop in China [1]. To increase the economic value of rapeseed, multi-purpose development and utilization strategy of rapeseed has been proposed with the goal of integrated use of oil, flower, vegetable, honey, feed, fertilizer in Chinese Rapeseed Industry [2]. The rapeseed sprout, as a vegetable, is an important measure to increase the value of rapeseed products.

In 1979, the Western Canadian oilseed Association registered the term “canola” to sell “double-low” rapeseed varieties with erucic acid (C22:1) of less than 5% and glucosinolates of less than 40 umol/g. Since “double low” rapeseed entered in the United States’ GRAS food list, there have been significant changes in fatty acid content of commercial rapeseed worldwide. In China, the double-low rapeseed has accounted for about 90% of commercial rapeseed by breeding and application of high-quality rapeseed varieties. At present, the commercial rapeseed components primarily consist of fatty acids and protein, of which fatty acids account for approximately 40–60% of the seed weight with rich unsaturated fatty acids such as oleic acid, linoleic acid, and linolenic acid [3]. Among these acids, oleic acid has been reported to reduce blood fat and prevent heart disease [4]. Linoleic acid plays an important role in softening blood vessels, lowering cholesterol levels, and preventing hypertension and heart disease [5,6]. Linolenic acid is a precursor of docosahexaenoic acid (DHA) and eicosapentaenoic acid (EPA), and it can reduce inflammation in cardiac fibrosis and hepatic steatosis in a high fat diet-induced metabolic syndrome rat model [7]. Protein is a critical indicator of food nutrition value, and it is primarily determined by the amino acid composition [8]. Rapeseed contains approximately 30% protein and has diverse amino acids. Furthermore, the proportion of essential amino acids in total amino acids in rapeseed conforms to the ideal protein distribution [9].

Rapeseed is rich in vitamin E, phytosterols, polyphenols, flavonoids, polypeptides, polysaccharides, and other active ingredients [2]. Vitamin E is an essential but not self-synthesized vitamin for humans and animals, and it is often obtained from food, with 70% derived from vegetable oil [10]. Vitamin E performs a variety of physiological functions, including anti-oxidation, cholesterol synthesis inhibition, and tumor cell growth inhibition [11,12]. Additionally, Vitamin E can be used to enhance sperm quality to treat miscarriage [11,13,14]. In cruciferous plants, glucosinolates are significant secondary metabolites [15,16]. The hydrolyzate of glucoraphanin in aliphatic glucosinolates exhibits significant anticancer activity [17]. 3-indole methanol, a hydrolysate of glucobrassicin in indole glucosinolates, has been reported to inhibit the proliferation of several types of tumor cells [18,19,20]. Rapeseed has diverse types of glucosinolates which vary greatly within rapeseed varieties, and thus rapeseed has great potential for genetic improvement [21]. Selenium, one of the essential elements for human body, is recognized as a “longevity element” [22]. Selenium-rich food is one of the main pathways for humans to obtain organic selenium. Rapeseed has been reported to have a special function to accumulate elemental selenium [23].

Sprouts are edible products that are grown directly from plant seeds or other nutrient storage organs under dark or light conditions [24], and they are also known as “living vegetables”. Sprouts, as nutrient-dense, high-quality, non-polluting green food, have a unique flavor and multiple health benefits, therefore they are preferred by consumers [25,26]. Sprouts contain more soluble sugar, chlorophyll, and carotenoids than seeds [27]. Additionally, germination can promote the accumulation of active ingredients [28,29]. For example, germination increases the content of amino acids, simple sugars, and other nutrients [30]. Moreover, germination also decreases the levels of anti-nutritional components and increases the digestibility and sensory attributes of sprouts [31]. Numerous studies have shown that the consumption of sprouts can effectively reduce the risk of diseases including intestinal disease, cardiovascular disease, diabetes, and cancer [32,33,34]. At present, bean sprouts including mung bean sprouts and soybean sprouts are the common vegetables consumed in China and many other countries [35].

Commodity rapeseed has significant health-promoting functions, however, there are a few reports on the nutrition evaluation of rapeseed sprouts, which limit the potential of rapeseed sprouts as a new type of healthy sprouts and further development of rapeseed industry. In this study, we compared the basic nutrients of eight rapeseed sprouts and five common bean sprouts, and analyzed the distribution of three dominant nutrients including vitamin E, glucosinolates, and selenium in seeds and sprouts of 44 rapeseed varieties. The objectives of this study are to: (1) Clarify the nutritional advantages of rapeseed sprouts by the comparison with bean sprouts; (2) analyze the transformation and accumulation of active components of rapeseed during germination; and (3) investigate the distribution of three dominant nutrients in rapeseed sprouts. This study will explore the nutritional advantages of rapeseed sprouts, provide reference for the commercial development of rapeseed sprouts, and lay theoretical basis for subsequent material screening and molecular polymerization breeding.

## 2. Materials and Methods

### 2.1. Plant Materials and Sample Treatments

Five common beans (*Vigna angularis*, *Vigna radiata*, *Pisum sativum*, *Glycine max*, and *Glycine max var*) and eight rapeseed (*Brassica napus*) varieties were employed to investigate the sprouts’ basic nutritional composition. For each legume crop, three to five varieties were used in this study, including four *Vigna angularis*, five *Vigna radiata*, three *Pisum sativum*, five *Glycine max*, and three *Glycine max var*. All the sprouts were germinated in the dark at a temperature of 20–25 °C, harvested at 5–7 cm in length, put into liquid nitrogen for quick freezing, ground into powder with a wall breaker, and then freeze-dried with a freeze dryer.

The seeds of these materials were obtained from Yangluo Experimental Base of Oil Crop Research Institute, Chinese Academy of Agricultural Sciences, Wuhan City, Hubei Province in 2019. During the entire growth process, only conventional field management was carried out. The 44 rapeseed varieties with a wide range of sources and extensive genetic backgrounds were selected for the subsequent determination of vitamin E, glucosinolate, and selenium contents.

### 2.2. Total Nutrient Composition Analysis

The nutrients contained in sprouts of five common beans and eight B. napus varieties including fructose, glucose, crude protein, crude fiber, vitamin E, minerals, fatty acids, amino acids, and glucosinolates, were tested by the Quality Inspection and Test Center for Oilseed Products, Ministry of Agriculture and Rural Affairs. The detection method of soluble sugar was determined by high-performance liquid chromatography in accordance with the Chinese agricultural industry standard GB/T 30390-2013. Fatty acid determination method: extract fat from vegetable sprouts with hydrolyzation-ether solution, saponification and methyl esterification under alkaline conditions to generate fatty acid methyl ester, capillary column gas chromatography analysis, quantitative determination of fatty acid methyl ester content. The method is based on the Chinese agricultural industry standard GB5009.168-2016.Amino acids were determined by referring to the Chinese agricultural industry standard GB 5009.124-2016: the protein in sprouts was hydrolyzed into free amino acids by hydrochloric acid, separated by an ion exchange column, and then reacted with ninhydrin solution to produce color reaction, and the amino acid content was determined by visible light spectrophotometry detector. All the data collected in this study were those of dry matter (DM) content.

### 2.3. Vitamin E Content Determination in Seeds and Sprouts

Vitamin E was extracted with n-hexane, and after leaching overnight, it was centrifuged, filtered, and determined on the Waters UPLC system. The mobile phase was prepared with 99% n-hexane and 1% isopropanol at a flow rate of 1 mL/min. The injection volume was set as 5 μL. The column temperature was controlled at 30 °C, and the UV detection wavelength was 292 nm. Vitamin E content was calculated using α-tocopherol as the equivalent. Based on the Chinese agricultural industry standard GB5009.82-2016, Vitamin E was calculated, and the calculation formula was as follows.

VE = α-T + 0.5 × β-T + 0.1 × γ-T + 0.01 × δ-T(1)

where α-T, β-T, γ-T, and δ-T represented α-tocopherol, β-tocopherol, γ-tocopherol, and δ-tocopherol, respectively.

### 2.4. Glucosinolate Content Determination in Seeds and Sprouts

The glucosinolate was determined using Waters UPLC system referring to the Chinese agricultural industry standard NY/T 1582-2007 and identified by comparing the retention time with standard one. The mobile phase consisted of phase A (ultrapure water) and phase B (20% acetonitrile in water). The glucosinolate determination was performed as follows. The injection volume was set as 10 μL, and the eluted desulphoglucosinolates were monitored at a flow rate of 0.55 mL/min and a wavelength of 229 nm. Desulphoglucosinolates in samples were quantified using relevant response factors and relative peak area.

### 2.5. Selenium Content Determination in Seeds and Sprouts

The selenium content in seeds and sprouts was determined according to the Chinese agricultural industry standard GB5009.93-2017 (Determination of Selenium in Foods of National Food Safety Standards). The samples were digested with a solution at 9:1 of nitric acid to perchloric acid, and the selenium content was determined using an atomic fluorescence spectrometer with 5% hydrochloric acid as carrier fluid and 2% potassium borohydride solution as reducing agent.

### 2.6. Statistical Analysis

The average values of each basic nutrient content (including sugar, crude protein, crude fiber, vitamin E, minerals, fatty acids, amino acids, and glucosinolates) in the investigated eight rapeseed sprout varieties and five species of bean sprout (four *Vigna angularis*, five *Vigna radiata*, three *Pisum sativum*, five *Glycine max*, and three *Glycine max var*) were subjected to statistical analysis, and their differences between rapeseed sprouts and bean sprouts were analyzed using SPSS 26.0 software (SPSS, Inc., Chicago, IL, USA) at a significance level of *p* < 0.05. Pearson correlation analyses of the three dominant nutrients (Vitamin E, glucosinolate, and selenium contents) in 44 rapeseed varieties between sprouts and seeds were performed. The variance, kurtosis, and skewness of the three dominant nutrients in 44 rapeseed varieties were also performed.

## 3. Results

### 3.1. Statistical Analyses of Basic Nutrients in Rapeseed Sprouts and Bean Sprouts

#### 3.1.1. Glucose, Crude Fiber, Crude Protein, and Fructose

**Figure 1:**
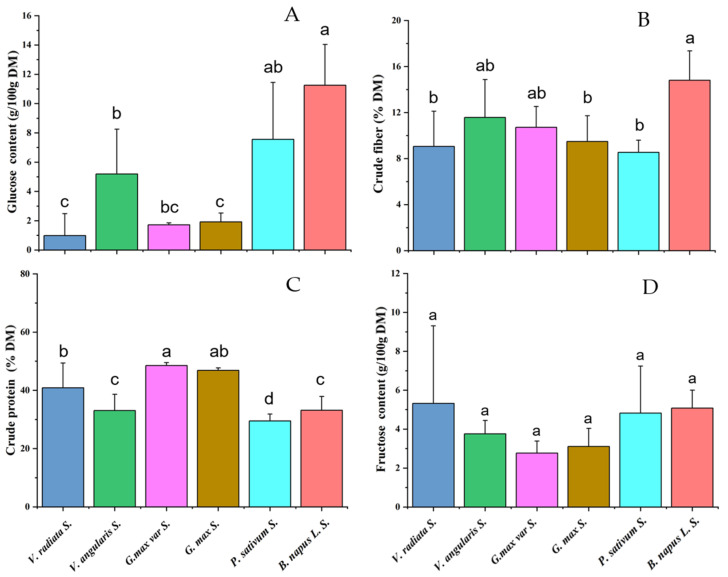
Comparison of fructose, glucose, crude protein, and crude fiber contents between rapeseed sprout and bean sprouts. (**A**) Glucose content. (**B**) Crude fiber content. (**C**) Crude protein content. (**D**) Fructose content. The different lowercase letters a, b, c, and d represent significant differences in investigated sprouts (*p* < 0.05).

As the basic nutrients of foods, the glucose, crude fiber, crude protein, and fructose contents in the dry matter of sprouts of five common bean species (each containing 3 to 5 varieties) and eight rapeseed varieties were determined. The statistical analysis showed that rapeseed sprouts contained significantly higher glucose content than the bean sprouts, except P. sativum (*p* < 0.05) (Figure 1A). Furthermore, the average crude fiber content in rapeseed sprouts was also higher than that in the bean sprouts, but only significantly higher than that in three bean species V. radiata, P. sativum, and G. max (*p* < 0.05) (Figure 1B). There was no significant difference in crude protein and fructose contents between rapeseed sprouts and bean sprouts (Figure 1C,D). Compared with bean sprouts, rapeseed sprouts exhibited advantages in glucose and crude fiber contents, but no significant advantage in the fructose and crude protein contents.

#### 3.1.2. Amino Acid Composition

As the basic component of protein, the composition and content of amino acids in food play an essential role in human health. We compared the distribution and content of every amino acid in the investigated sprouts. As shown in Table 1, rapeseed sprouts had no obvious nutritional advantage and disadvantage over the five bean sprouts in terms of total amino acids and amino acid composition. However, essential amino acids (EAA) in rapeseed sprouts accounted for 33% of total amino acids (TAA). The ratio of essential amino acids (EAA) to non-essential amino acids (NEAA) was 49%. The values of EAA/TAA and EAA/NEAA of the rapeseed sprout were highest among the investigated sprouts, close to the 40% and above 60% recommended by FAO/WHO, suggesting that rapeseed sprout was a relatively high-quality protein source [36].

#### 3.1.3. Fatty Acids

Fatty acids are the important component of rapeseed, accounting for 40–60% of seed weight in high-quality rapeseed. This study investigated the composition of fatty acids in these sprouts, especially the proportion of unsaturated fatty acids such as oleic, linoleic, and linolenic acids. Compared with bean sprouts, rapeseed sprouts exhibited the highest percentage of unsaturated fatty acids (90.34%) in total fatty acids, and the lowest percentage of saturated fatty acid (9.66%) (Table 2). The saturated fatty acid percentage of five bean sprouts ranged from 16.67% to 40.47%. In addition, the oleic acid percentage of rapeseed sprouts reached as high as 48.11% (Table 2). Overall, our data showed that rapeseed sprouts displayed a lower saturated fatty acid proportion and a more balanced unsaturated fatty acid composition than bean sprouts, thus rapeseed sprouts could better meet the needs of human health.

#### 3.1.4. Minerals

**Figure 2:**
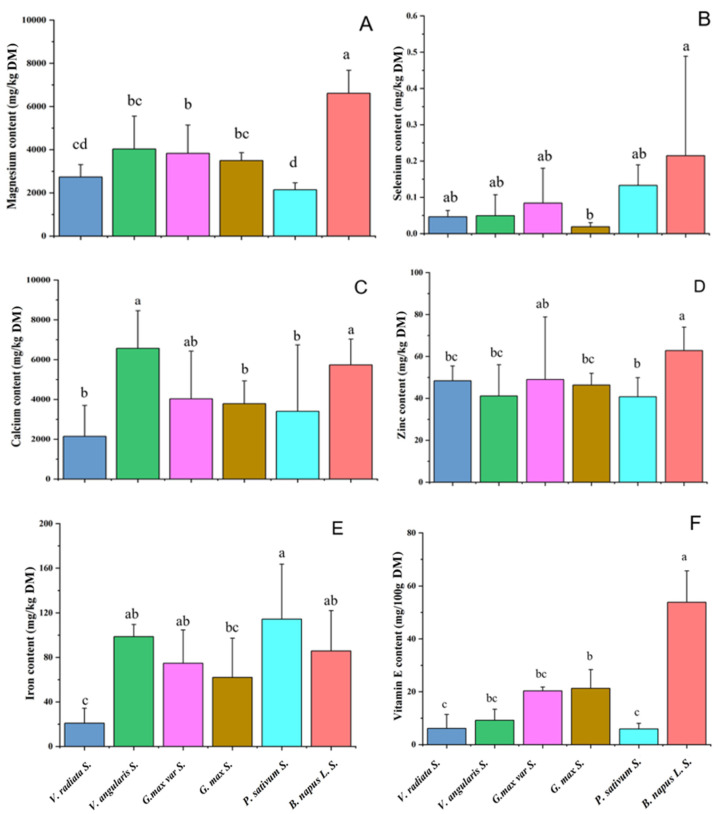
Comparison of five mineral element contents (magnesium, selenium, calcium, zinc, and iron) and Vitamin E content in investigated sprouts. (**A**) Magnesium content. (**B**) Selenium content. (**C**) Calcium content. (**D**) Zinc content. (**E**) Iron content. (**F**) Vitamin E content. The different lowercase letters a, b, c, and d represent significant differences in investigated sprouts (*p* < 0.05).

Minerals are essential for building body tissues and maintaining normal physiological functions, and they could be obtained from food and drinking water. In this study, five important mineral element contents in the sprouts including magnesium, selenium, calcium, zinc, and iron concentrations were determined. As shown in Figure 2A, the average magnesium content in rapeseed sprouts was significantly higher than that in the bean sprouts (*p <* 0.05). The average selenium content in rapeseed sprouts was also higher than that in bean sprouts, but this difference was not significant due to the large variation across rapeseed varieties (Figure 2B). In addition, no significant differences in the contents of calcium, zinc, and iron were observed between rapeseed sprouts and bean sprouts (Figure 2C–E). The results indicated that rapeseed sprouts were rich in minerals and had certain advantages in magnesium and selenium contents over bean sprouts.

#### 3.1.5. Vitamin E

The difference in average VE content in eight rapeseed sprouts and five bean sprouts was analyzed using SPSS software. As shown in Figure 2F, the average VE content in rapeseed sprouts was 2.5–9 times as high as that in bean sprouts (*p* < 0.05), indicating that rapeseed sprouts had significant advantages over bean sprouts in terms of VE content.

#### 3.1.6. Glucosinolates

**Figure 3:**
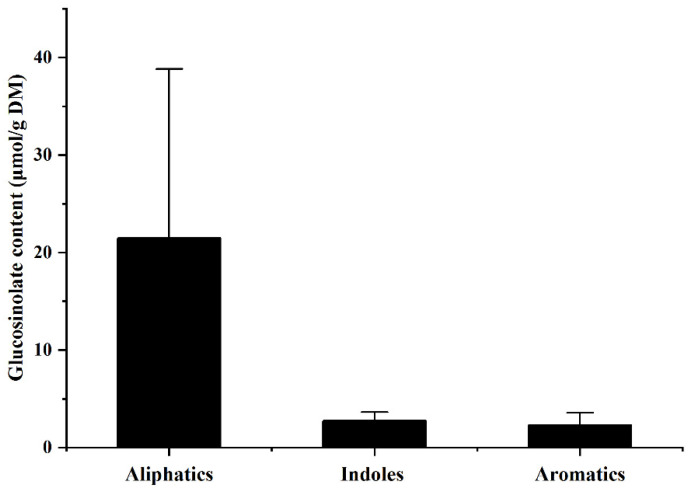
Contents of glucosinolate types in rapeseed sprouts.

Glucosinolate is a sulfur-containing secondary metabolite found exclusively in the Cruciferae [37]. Some glucosinolates, especially glucoraphanin and glucobrassicin, have remarkable anticancer activity [38]. In terms of the peak area, the glucosinolate content in all the investigated bean sprouts was negligibly low, while its average content in rapeseed sprouts was up to 26.96 umol/g, which was mainly composed of aliphatic glucosinolates (Figure 3). Large variation in glucosinolate content was observed across *B. napus L.* varieties. These results suggested the advantages of rapeseed sprouts in providing glucosinolates with anti-cancer properties.

### 3.2. Comparison of Dominant Nutrients in Different Varieties of Rapeseed Sprouts

Rapeseed sprouts outperformed five bean sprouts in terms of vitamin E, glucosinolate, and selenium contents. These demonstrated that rapeseed sprout not only possessed the basic properties as common vegetables, but also had the potential to be developed into functional vegetables. Thus, this study determined the contents of vitamin E, glucosinolate, and selenium in 44 rapeseed sprouts varieties with the purpose of establishing a scientific basis for the development and utilization of rapeseed sprouts.

#### 3.2.1. Determination of Vitamin E Content

By measuring the vitamin E content in 44 *B. napus L*. seeds and sprouts (Table 3 and Table S1), we found that the rapeseed seeds were dominated by γ-tocopherol with the average α-tocopherol/γ-tocopherol ratio of 0.53, whereas rapeseed sprouts were dominated by α-tocopherol with the average α-tocopherol/γ-tocopher ratio of 9.65, which was about 18.20 times as high as that in seeds. The average content of α-tocopherol and γ-tocopherol in rapeseed sprouts was 47.90 mg/100 g and 9.25 mg/100 g, respectively with the corresponding variation coefficient of 25.47% and 130.33%. However, the average content of α-tocopherol and γ-tocopherol in seeds was respectively 25.76 mg/100 g and 47.37 mg/100 g with the corresponding variation coefficient of 60.56% and 57.40%. Furthermore, the correlation coefficients in vitamin E content and α-tocopherol content between seeds and sprouts were 0.425 and 0.451, respectively (*p* < 0.05), showing significantly positive correlations. In conclusion, α-tocopherol and γ-tocopherol contents of rapeseed had a wide range of variation in both seeds and sprouts, and thus rapeseed had a great potential for genetic improvement. Our data indicated that germination promoted the conversion from γ-tocopherol in seeds into α-tocopherol in sprouts.

#### 3.2.2. Determination of Glucosinolate Content

The glucosinolate determination in the 44 *B. napus L.* varieties showed that the content of glucosinolate in rapeseed sprouts depended on the content of glucosinolate in rapeseed seeds with the correlation coefficient of 0.748 (*p* < 0.01). As shown in Table 4 and Table S2, germination increased not only the total amount of glucosinolates, but also the types of glucosinolates. Rapeseed sprouts contained a total of 12 types of glucosinolates consisting of 6 aliphatic glucosinolates, 4 indole glucosinolates, and 2 aromatic glucosinolates. However, rapeseed seeds contained a total of 10 types of glucosinolates composed of 5 aliphatic glucosinolates, 3 indole glucosinolates, and 2 aromatic glucosinolates. In rapeseed sprouts, the glucosinolate content varied greatly with types, of which the average contents of progoitrin (PRO), 4-hydroxyglucobrassicin (4OH), and gluconapin (NAP) were relatively high, accounting for 39.07%, 11.29%, and 20.45% in the total glucosinolate. In rapeseed sprouts, the average contents of epi-progoitrin (EPRO) and neoglucobrassicin (NEO) were relatively low, accounting for 0.85% and 0.31% in the total glucosinolates. EPRO and NEO were observed in sprouts, but not in seeds. During the germination process, the contents of NAP and PRO hardly changed, whereas the contents of glucoraphanin (RAA), gluconapoleiferin (GAL), glucobrassicanapin (GBN), and glucotropaeolin (TRO) increased. The proportion of aliphatic glucosinolate and aromatic glucosinolate increased from 63.79% and 10.33% in seeds to 65.32% and 17.33% in sprouts, while the proportion of indole glucosinolate decreased from 25.88% in seeds to 18.04% in sprouts. In particular, RAA, a precursor of the anticancer compound sulforaphane, increased from 1.06% to 1.62% when seeds germinated into sprouts. These results suggested the possible morphological transformation of glucosinolates during rapeseed germination, even the accumulation of some anticancer glucosinolates.

#### 3.2.3. Determination of the Selenium Content

We investigated the selenium content in seeds and sprouts in 44 *B. napus L*. varieties. The results showed that the selenium contents ranged from 33.25 µg/kg to 78.62 µg/kg in rapeseed sprouts with an average value of 52.86 µg/kg, and from 23.99 µg/kg to 67.44 µg/kg in the seeds with an average of 42.00 µg/kg, and the kurtosis and skewness in both sprouts and seeds were less than 1 (Table 5 and Table S3). The selenium content was slightly higher in rapeseed sprouts than in seeds with extremely significant correlation between sprouts and seeds (r = 0.704, *p* < 0.01). The selenium content in rapeseed sprouts was higher than the reported average selenium content in crops and foods [39,40], indicating its advantages as a selenium supplementation food. Furthermore, the selenium content in rapeseed displayed considerable variations, suggesting a potential of rapeseed selenium biofortification.

## 4. Discussion

### 4.1. Analysis of Basic Nutrients in Rapeseed and Bean Sprouts

The comparison of basic nutrients between rapeseed sprouts and bean sprouts revealed that rapeseed sprouts had obvious advantages in the contents of glucose and crude fiber over bean sprouts. The soluble sugar content in rapeseed sprouts was about 16 mg/100 g, which was close to that in organic vegetables [41]. The crude fiber content (13.09% in dry matter) in rapeseed sprouts was significantly higher than that in bean sprouts, and rapeseed sprouts also exhibited obvious advantages in crude fiber content over celery (0.87%) which was rich in crude fiber in vegetables [42]. This makes it possible to meet the demands of urban inhabitants whose diets are dominated by refined food for acquire crude fiber.

Our data showed that essential amino acids (EAA) accounted for 33% in total amino acids (TAA) in rapeseed sprouts, and the ratio of EAA to non-essential amino acids (NEAA) was 49%, which was close to the FAO/WHO recommendations of 40% and above 60% [36]. Since it contained all of the essential amino acids, the rapeseed sprout could be an excellent source of amino acids and have a significant impact on the nutritional quality of plant-based products. Umami amino acids include aspartic acid, glutamic acid, glycine, phenylalanine, alanine, and tyrosine [43]. The presence of these amino acids endows vegetables with a distinct umami flavor. Rapeseed sprouts were rich in aspartic and glutamic acids, thus contributing to the sweet flavor.

Minerals are critical for human physical and mental health to maintain normal life activities [44]. Detection of the mineral levels of rapeseed sprouts will provide a scientific foundation for optimizing the mineral element composition of rapeseed sprouts, thereby improving the nutrition of rapeseed sprouts to satisfy human’s needs. The calcium and magnesium contents in investigated rapeseed sprouts were up to 6838 mg/Kg and 5946 mg/Kg, respectively. The contents of iron, zinc, and selenium in the rapeseed sprouts precisely satisfied the human requirements according to the scientific and safe trace element ingestion range. It is particularly worth noting that the rapeseed sprouts are rich in selenium, and selenium has become a research hot topic in recent years. Selenium has been reported to have multiple health-promotion functions such as lowering blood triglycerides, boosting intestinal microbiota health, enhancing male reproductive health, and preventing cancer [45]. Our results indicated that the rapeseed sprout could be a desirable mineral source.

### 4.2. Dominant Nutrients in Rapeseed Sprouts

Vitamin E is composed of eight isomers, of which the most active one is α-tocopherol [46]. Our survey found that γ-tocopherol was the main vitamin E in rapeseed, followed by α-tocopherol, which was similar to the content distribution of vitamin E in rapeseed reported by previous studies [47]. However, the rapeseed sprout was dominated by α-tocopherol, whereas γ-tocopherol was present in small amount. Based on these results, we speculated that rapeseed might undergo the conversion from γ-tocopherol to α-tocopherol during germination.

Cruciferous plants are acknowledged as “natural glucosinolate synthesizers” [17]. A large number of studies have reported that cruciferous vegetables can effectively prevent cancer [20,48,49]. Long-term consumption of Brassica vegetables can dramatically reduce the risk of cancers such as gastric cancer, rectal cancer, and colon cancer [50]. Germination induced the change in the proportion of glucosinolates in rapeseed: aliphatic glucosinolates increased from 63.79% to 65.32%, indole glucosinolates decreased from 25.88% to 17.34%, and aromatic glucosinolates increased from 10.33% to 17.33%. Based on these results, we speculated that glucosinolates in rapeseed might undergo transformation and accumulation during the germination process. Compared to seeds, rapeseed sprouts contained higher percentage of RAA with obvious anti-cancer properties. Considering this, we inferred that germination of rapeseed might contribute to the transformation and accumulation of active substances, making rapeseed sprouts more beneficial to human health.

Selenium, as an essential nutrient, is involved in a wide variety of physiological functions [51]. Due to the unique role of selenium and widespread selenium insufficiency worldwide, selenium supplementation through foods has become a critical means to promote human health [52]. Our investigation revealed that rapeseed had certain advantages in selenium enrichment, compared with beans. In addition, the selenium content in rapeseed sprouts and seeds had considerable variations across varieties, indicating its great potential for development and utilization.

It has been reported that the combination of selenium and vitamin E has a greater effect on improving animal immunity and fecundity than their application alone, particularly on improving male sperm quality and female ovary development [53]. In our study, rapeseed sprout was rich in both selenium and vitamin E, which could simultaneously meet the demand for these two nutrients by humans. Considering its rich nutrition and active ingredients, rapeseed sprout is suitable to be developed into a functional vegetable as the daily diet. The further development and utilization of the vegetable functions of rapeseed will add to the value of rapeseed and contribute to the development of rapeseed industry.

## 5. Conclusions

By comparing the nutrients of eight rapeseed sprouts and five kinds of common bean sprouts (each sprout contains 3–5 varieties), we found that rapeseed sprouts were rich in glucose, VE, crude fiber, selenium, magnesium, and glucosinolates. Further examination of the nutritional composition of various rapeseed varieties revealed that germination induced the transformation and accumulation of the most physiologically active α-tocopherol in the rapeseed sprouts, potentially enhancing their anti-oxidant effects. During seed germination, the percentage of RAA, a glucosinolate with obvious anticancer effects, was increased. The transformation of tocopherol and glucosinolates demonstrated that germination was conducive to the conversion and accumulation of active substances. Three dominant nutrients (vitamin E, glucosinolates, and selenium) found in rapeseed sprouts, are quantitative genetic substances, and thus they are suitable for subsequent material screening and molecular polymerization. Our study indicates that rapeseed sprouts have the potential to further strengthen and utilize the active ingredients in rapeseed, thereby increasing the additional value of rapeseed commodities and providing consumers with functional nutritious vegetable.

## Figures and Tables

**Table 1:** Amino acid distribution of sprouts.

Amino Acid	*V. Radiata S.*		*V. Angularis S.*		*G. Max Var S.*		*G. Max S.*		*P. Sativum S.*		*B. Napus L.S.*	
	Mean	SD	Mean	SD	Mean	SD	Mean	SD	Mean	SD	Mean	SD
Asp	3.76	1.32	3.60	0.91	8.24	1.31	6.58	1.84	2.90	0.36	1.81	0.22
Thr	1.02	0.30	0.71	0.01	1.28	0.07	1.18	0.07	0.65	0.05	1.03	0.08
Ser	1.32	0.37	0.84	0.10	1.59	0.14	1.54	0.13	0.86	0.04	0.89	0.07
Glu	5.41	1.02	2.23	0.33	4.12	0.66	4.34	0.70	3.09	0.34	3.91	0.91
Gly	0.97	0.32	0.61	0.06	1.17	0.12	1.15	0.13	0.65	0.05	0.93	0.08
Ala	1.13	0.30	0.73	0.10	1.30	0.09	1.28	0.08	0.77	0.07	0.96	0.09
Val	0.10	0.07	0.04	0.01	0.17	0.02	0.14	0.02	0.06	0.01	0.08	0.02
Met	1.79	0.20	1.32	0.07	1.94	0.05	1.84	0.05	1.09	0.08	1.44	0.15
Ile	0.20	0.04	0.17	0.03	0.25	0.04	0.21	0.04	0.10	0.02	0.25	0.04
Leu	1.17	0.21	0.82	0.04	1.28	0.09	1.27	0.06	0.65	0.05	0.83	0.08
Trp	0.64	0.23	0.68	0.10	1.14	0.08	1.09	0.09	0.47	0.04	0.75	0.12
Phe	1.50	0.26	0.97	0.11	1.67	0.12	1.66	0.07	0.85	0.06	0.84	0.06
Lys	1.77	0.35	1.24	0.13	2.02	0.15	1.97	0.18	1.18	0.09	1.56	0.10
His	0.93	0.16	0.66	0.04	1.08	0.05	0.96	0.07	0.57	0.03	0.73	0.10
Arg	1.92	0.45	1.13	0.11	2.42	0.17	2.31	0.15	1.45	0.26	1.48	0.29
Pro	0.90	0.20	0.63	0.07	1.02	0.11	0.98	0.11	0.52	0.05	0.85	0.12
TAA	24.54	5.72	16.38	0.33	30.69	0.46	28.51	0.85	15.87	1.25	18.34	2.28
EAA	7.55	1.43	5.26	0.40	8.60	0.53	8.27	0.49	4.59	0.37	6.03	0.53
NEAA	16.99	4.36	11.13	1.83	22.09	2.72	20.24	3.31	11.28	1.24	12.31	2.00
EAA/NEAA	0.44		0.47		0.39		0.41		0.41		0.49	
EAA/TAA	0.31		0.32		0.28		0.29		0.29		0.33	

Notes: TAA, total amino acids; EAA, essential amino acids (Lys, Phe, Met, Thr, Leu, Ile, Val and Trp); NEAA, non-essential amino acids.

**Table 2:** Fatty acid content of sprouts.

Types of Sprouts	Saturated Fatty Acid (%)		Unsaturated Fatty Acids (%)		Oleic (%)		Linoleic (%)		Linolenic (%)	
	Mean	SD	Mean	SD	Mean	SD	Mean	SD	Mean	SD
*V. radiata S.*	23.56	8.19	76.43	8.19	12.65	4.00	42.25	14.78	21.09	10.23
*V. angularis S.*	40.47	7.60	59.85	7.60	4.83	1.67	30.90	2.88	22.77	7.31
*G. max var S.*	16.67	0.26	83.33	0.26	16.15	0.64	52.50	0.55	14.30	0.90
*G. max S.*	17.33	0.90	82.67	0.90	14.89	1.04	55.65	1.91	11.65	1.08
*P. sativum S.*	24.25	4.23	75.83	4.23	9.42	3.03	52.47	5.27	12.88	4.79
*B. napus L.S.*	9.66	1.80	90.34	1.80	48.11	7.90	21.39	3.50	15.02	2.25

**Table 3:** Distribution of tocopherol in rapeseed sprouts and seeds.

		Mean (mg/100 g)	Max (mg/100 g)	Mini (mg/100 g)	SD	CV%
Sprouts	α-T	47.90	76.29	14.11	12.20	25.47
	γ-T	9.25	79.69	2.48	12.06	130.33
	VE	48.83	76.55	15.31	12.29	25.17
	α-T/γ-T	9.65	30.09	0.62	6.38	66.09
Seeds	α-T	25.76	61.61	0.47	13.84	53.71
	γ-T	47.37	111.51	3.59	23.31	49.22
	VE	30.50	72.76	0.83	15.86	52.02
	α-T/γ-T	0.53	0.86	0.13	0.15	26.61

**Table 4:** Distribution of glucosinolate in rapeseed sprouts and seeds.

		Sprouts			Seeds		
		Mean (µmol/g)	Range (µmol/g)	SD	Mean (µmol/g)	Range (µmol/g)	SD
Aliphatic Glucosinolates	Progoitrin (PRO)	12.16	0~71.10	19.36	6.22	0~38.03	9.68
		39.07%			38.59%		
	Epi-progoitrin (EPRO)	0.27	0~4.30	0.72	-	-	-
		0.85%					
	Glucoraphanin (RAA)	0.50	0~3.19	0.20	0.17	0~1.53	0.53
		1.62%			1.06%		
	Gluconapoleiferin (GAL)	0.44	0~1.64	0.37	0.14	0~0.75	0.22
		1.41%			0.84%		
	Gluconapin (NAP)	6.36	0.90~19.03	3.90	3.59	0.25~19.62	5.19
		20.45%			22.25%		
	Glucobrassicanapin (GBN)	0.60	0~1.90	0.51	0.20	0~1.88	0.66
		1.92%			1.05%		
Indole glucosinolates	4-hydroxyglucobrassicin (4OH)	3.51	0~13.27	3.79	3.69	0~12.07	1.61
		11.29%			22.88%		
	Neoglucobrassicin (NEO)	0.10	0~1.50	0.35	-	-	-
		0.31%					
	Glucobrassicin (GBC)	0.43	0~3.20	0.63	0.27	0~5.45	1.12
		1.38%			1.68%		
	4-methoxyglucobrassicin (4ME)	1.36	11.03	2.56	0.21	0~1.02	0.22
		4.36%			1.32%		
Aromatic Glucosinolates	Glucotropaeolin (TRO)	3.97	0~9.66	3.28	0.15	0~4.10	1.32
		12.76%			0.95%		
	Gluconasturtiin (NAS)	1.42	0~11.51	3.07	1.51	0.13~12.95	1.90
		4.57%			9.38%		
Total		31.11	100.11	20.34	16.11	64.22	14.87

**Table 5:** Selenium content in rapeseed sprouts and seeds.

	Mean (μg/kg)	Mini (μg/kg)	Max (μg/kg)	Kurtosis	Skewness
Sprouts	52.86	33.25	78.62	0.48	0.70
Seeds	42.00	23.99	67.44	1.00	0.84

## Data Availability

The datasets generated for this study are available on request to the corresponding author.

## Supplementary Materials

The following supporting information can be downloaded at: https://www.mdpi.com/article/10.3390/foods11060778/s1, Table S1: The original data of vitamin E contents in seeds and sprouts of 44 rapeseed varieties, Table S2: The original data of glucosinolate contents in seeds and sprouts of 44 rapeseed varieties, Table S3: The original data of selenium contents in seeds and sprouts of 44 rapeseed varieties.

## References

[1] Hu D., Jing J., Snowdon R.J., Mason A.S., Shen J., Meng J., Zou J. (2021). Exploring the gene pool of Brassica napus by genomics-based approaches. Plant Biotechnol. J..

[2] Hangzhong W. (2018). Rapeseed industry development strategy oriented by new demand. Chin. J. Oil Crop Sci..

[3] Cui Y., Zeng X., Xiong Q., Wei D., Liao J., Xu Y., Chen G., Zhou Y., Dong H., Wan H. (2021). Combining quantitative trait locus and co-expression analysis allowed identification of new candidates for oil accumulation in rapeseed. J. Exp. Bot..

[4] Parzych K.R., Klionsky D.J. (2014). An overview of autophagy: Morphology, mechanism, and regulation. Antioxid. Redox Signal..

[5] Jandacek R.J. (2017). Linoleic acid: A nutritional quandary. Healthcare.

[6] Wannamethee S.G., Jefferis B.J., Lennon L., Papacosta O., Whincup P.H., Hingorani A.D. (2018). Serum conjugated linoleic acid and risk of incident heart failure in older men: The British regional heart study. J. Am. Heart Assoc..

[7] Leikin-Frenkel A.I. (2016). Is there a role for alpha-linolenic acid in the fetal programming of health?. J. Clin. Med..

[8] Møller A., Hammershøj M., Dos Passos N., Tanambell H., Stødkilde L., Ambye-Jensen M., Danielsen M., Jensen S., Dalsgaard T. (2021). Biorefinery of green biomass—How to extract and evaluate high quality leaf protein for food?. J. Agric. Food Chem..

[9] Wu J., Xu F., Wu Y., Xiong W., Pan M., Zhang N., Zhou Q., Wang S., Ju X., Wang L. (2020). Characterization and analysis of an oil-in-water emulsion stabilized by rapeseed protein isolate under pH and ionic stress. J. Sci. Food Agric..

[10] Vallibhakara S.A., Nakpalat K., Sophonsritsuk A., Tantitham C., Vallibhakara O. (2021). Effect of vitamin E supplement on bone turnover markers in postmenopausal osteopenic women: A double-blind, randomized, placebo-controlled trial. Nutrients.

[11] Violi F., Nocella C., Loffredo L., Carnevale R., Pignatelli P. (2021). Interventional study with vitamin E in cardiovascular disease and meta-analysis. Free Radic. Biol. Med..

[12] Khalid A., Bhuvanendran S., Magalingam K., Ramdas P., Kumari M., Radhakrishnan A. (2021). Clinically relevant genes and proteins modulated by tocotrienols in human colon cancer cell lines: Systematic scoping review. Nutrients.

[13] Abdel-Wahab A., Hassanin K., Mahmoud A., Abdel-Badeea W., Abdel-Razik A., Attia E., Abdelmohsen U., Abdel Aziz R., Najda A., Alanazi I. (2021). Physiological roles of red carrot methanolic extract and vitamin E to abrogate cadmium-induced oxidative challenge and apoptosis in rat testes: Involvement of the Bax/Bcl-2 ratio. Antioxidants.

[14] Hafez S., Elbassuoni E., Abdelzaher W., Welson N., Batiha G., Alzahrani K., Abdelbaky F. (2021). Efficacy of vitamin E in protection against methotrexate induced placental injury in albino rats. Biomed. Pharmacother. Biomed. Pharmacother..

[15] Merinas-Amo T., Lozano-Baena M., Obregón-Cano S., Alonso-Moraga Á., de Haro-Bailón A. (2021). Brassica rapaRole of glucosinolates in the nutraceutical potential of selected cultivars of. Foods.

[16] Liu Y., Zhou X., Yan M., Wang P., Wang H., Xin Q., Yang L., Hong D., Yang G. (2020). Fine mapping and candidate gene analysis of a seed glucosinolate content QTL, qGSL-C2, in rapeseed (*Brassica napus* L.). Theor. Appl. Genet..

[17] Fahey J., Zalcmann A., Talalay P. (2001). The chemical diversity and distribution of glucosinolates and isothiocyanates among plants. Phytochemistry.

[18] Salem A., Medhat D., Fathy S., Mohamed M., El-Khayat Z., El-Daly S. (2021). Indole glucosinolates exhibit anti-inflammatory effects on Ehrlich ascites carcinoma cells through modulation of inflammatory markers and miRNAs. Mol. Biol. Rep..

[19] Almuhayawi S., Almuhayawi M., Al Jaouni S., Selim S., Hassan A. (2021). BrassicaEffect of laser light on growth, physiology, accumulation of phytochemicals, and biological activities of sprouts of three cultivars. J. Agric. Food Chem..

[20] Lee Y.R., Chen M., Lee J.D., Zhang J., Lin S.Y., Fu T.M., Chen H., Ishikawa T., Chiang S.Y., Katon J. (2019). Reactivation of PTEN tumor suppressor for cancer treatment through inhibition of a MYC-WWP1 inhibitory pathway. Science.

[21] Tan Z., Xie Z., Dai L., Zhang Y., Hu Z., Tang S., Wan L., Yao X., Guo L., Hong D. (2022). Genome- and transcriptome-wide association studies reveal the genetic basis and the breeding history of seed glucosinolate content in Brassica napus. Plant Biotechnol. J..

[22] Plummer J., Postnikoff S., Tyler J., Johnson J. (2021). Selenium supplementation inhibits IGF-1 signaling and confers methionine restriction-like healthspan benefits to mice. eLife.

[23] Wang Y., Jia W., Hu C., Chen X., Wu Z., Zhao X. (2018). Characteristics of selenium enrichment in rape and its relationship with soil selenium. Acta Sci. Circumst..

[24] Le L., Gong X., An Q., Xiang D., Zou L., Peng L., Wu X., Tan M., Nie Z., Wu Q. (2021). Quinoa sprouts as potential vegetable source: Nutrient composition and functional contents of different quinoa sprout varieties. Food Chem..

[25] Colonna E., Rouphael Y., Barbieri G., De Pascale S. (2016). Nutritional quality of ten leafy vegetables harvested at two light intensities. Food Chem..

[26] Geng J., Li J., Zhu F., Chen X., Du B., Tian H., Li J. (2021). Plant sprout foods: Biological activities, health benefits, and bioavailability. J. Food Biochem..

[27] Li X., Li S., Lou H., Deng Z., Zhan X. (2019). Research progress of active ingredients in sprouts. Cereals Oils.

[28] Li J., Lu Y., Chen H., Wang L., Wang S., Guo X., Cheng X. (2021). Effect of photoperiod on vitamin E and carotenoid biosynthesis in mung bean (*Vigna radiata*) sprouts. Food Chem..

[29] Sharma S., Singh A., Singh B. (2019). Characterization of in vitro antioxidant activity, bioactive components, and nutrient digestibility in pigeon pea (*Cajanus cajan*) as influenced by germination time and temperature. J. Food Biochem..

[30] Liu H.K., Kang Y.F., Zhao X.Y., Liu Y.P., Zhang X.W., Zhang S.J. (2019). Effects of elicitation on bioactive compounds and biological activities of sprouts. J. Funct. Foods.

[31] Mir S.A., Farooq S., Shah M.A., Sofi S.A., Khaneghah A.M. (2021). An overview of sprouts nutritional properties, pathogens and decontamination technologies. LWT Food Sci. Technol..

[32] Teixeira-Guedes C.I., Oppolzer D., Barros A.I., Pereira-Wilson C. (2019). Phenolic rich extracts from cowpea sprouts decrease cell proliferation and enhance 5-fluorouracil effect in human colorectal cancer cell lines. J. Funct. Foods.

[33] Tang D., Dong Y., Ren H., Li L., He C. (2014). A review of phytochemistry, metabolite changes, and medicinal uses of the common food mung bean and its sprouts (*Vigna radiata*). Chem. Cent. J..

[34] Prakash D., Upadhyay G., Singh B.N., Singh H.B. (2007). Antioxidant and free radical-scavenging activities of seeds and agri-wastes of some varieties of soybean (*Glycine max*). Food Chem..

[35] Wei Y., Wang X., Shao X., Xu F., Wang H. (2019). Sucrose treatment of mung bean seeds results in increased vitamin C, total phenolics, and antioxidant activity in mung bean sprouts. Food Sci. Nutr..

[36] Wang Y., Yu S., Ma G., Chen S., Shi Y., Yang Y. (2014). Comparative study of proximate composition and amino acid in farmed and wild Pseudobagrus ussuriensis muscles. Int. J. Food Sci. Technol..

[37] Ouassou M., Mukhaimar M., El Amrani A., Kroymann J., Chauveau O. (2019). Biosynthesis of indole glucosinolates and ecological role of secondary modification pathways. Compt. Rend. Biol..

[38] Park M.H., Arasu M.V., Park N.Y., Choi Y.J., Kim S.J. (2013). Variation of glucoraphanin and glucobrassicin: Anticancer components in Brassica during processing. Food Sci. Technol..

[39] Chen L., Yang F., Xu J., Hu Y., Hu Q., Zhang Y., Pan G. (2002). Determination of selenium concentration of rice in china and effect of fertilization of selenite and selenate on selenium content of rice. J. Agric. Food Chem..

[40] You-Kai X.U., Liu H.M., Xiao C.F., Chan Y., Zhao-Lu W.U., Xiang-Sheng D., Cai C.T. (2004). An analysis of selenium contents of wild vegetable in xishuangbanna. Acta Bot. Yunnanica.

[41] Ma C., Li X., Ban T., Liang D. (2021). Analysis and comparison of nutrient quality between organic and conventional vegetables. Crops.

[42] Zhou Z. (2019). Determination of crude fiber content in 6 kinds of common vegetables. Mod. Hortic..

[43] Zhang Y., Pan Z., Venkitasamy C., Ma H., Li Y. (2015). Umami taste amino acids produced by hydrolyzing extracted protein from tomato seed meal. LWT Food Sci. Technol..

[44] Angeli V., Miguel Silva P., Crispim Massuela D., Khan M., Hamar A., Khajehei F., Graeff-Hönninger S., Piatti C. (2020). *Chenopodium quinoa* Quinoa (Willd.): An overview of the potentials of the “golden grain” and socio-economic and environmental aspects of its cultivation and marketization. Foods.

[45] Ren X., Wang S., Zhang C., Hu X., Zhou L., Li Y., Xu L. (2020). Selenium ameliorates cadmium-induced mouse leydig TM3 cell apoptosis via inhibiting the ROS/JNK/c-jun signaling pathway. Ecotoxicol. Environ. Safety.

[46] Saito Y. (2021). Diverse cytoprotective actions of vitamin E isoforms- role as peroxyl radical scavengers and complementary functions with selenoproteins. Free Rad. Biol. Med..

[47] Fritsche S. (2012). A candidate gene-based association study of tocopherol content and composition in rapeseed (*Brassica napus*). Front. Plant Sci..

[48] Melchini A., Traka M.H., Catania S., Miceli N., Costa C. (2013). Antiproliferative activity of the dietary isothiocyanate erucin, a bioactive compound from cruciferous vegetables, on human prostate cancer cells. Nutr. Cancer. Int. J..

[49] Sapone A., Affatato A., Canistro D., Pozzetti L., Broccoli M., Barillari J., Iori R., Paolini M. (2007). Cruciferous vegetables and lung cancer. Mutat. Res..

[50] Fahey J., Wehage S., Holtzclaw W., Kensler T., Egner P., Shapiro T., Talalay P. (2012). Protection of humans by plant glucosinolates: Efficiency of conversion of glucosinolates to isothiocyanates by the gastrointestinal microflora. Cancer Prev. Res..

[51] Schwarz K., Foltz C. (1999). Selenium as an integral part of factor 3 against dietary necrotic liver degeneration. 1951. Nutrition.

[52] Bhatia P., Aureli F., D’Amato M., Prakash R., Cameotra S.S., Nagaraja T.P., Cubadda F. (2013). Selenium bioaccessibility and speciation in biofortified Pleurotus mushrooms grown on selenium-rich agricultural residues. Food Chem..

[53] Wuryastuti H., Stowe H.D., Bull R.W., Miller E.R. (1993). Effects of vitamin E and selenium on immune responses of peripheral blood, colostrum, and milk leukocytes of sows. J. Anim. Sci..

